# Reliable and cost effective design of intermetallic Ni_2_Si nanowires and direct characterization of its mechanical properties

**DOI:** 10.1038/srep15050

**Published:** 2015-10-12

**Authors:** Seung Zeon Han, Joonhee Kang, Sung-Dae Kim, Si-Young Choi, Hyung Giun Kim, Jehyun Lee, Kwangho Kim, Sung Hwan Lim, Byungchan Han

**Affiliations:** 1 Structural Materials Division, Korea Institute of Materials Science, Changwon 642-831, Republic of Korea; 2 Department of Energy Systems Engineering, DGIST, Daegu, 0711-873, Republic of Korea; 3 Gangwon Regional Division, Korea Institute of Industrial Technology, Gangneung 210-340, Republic of Korea; 4 Department of Materials Science and Engineering, Changwon National University, Changwon 641-773, Republic of Korea; 5 School of Materials Science and Engineering, Pusan National University, Busan, 609-735, Republic of Korea; 6 Department of Advanced Materials Science and Engineering, Kangwon National University, Chuncheon 200-701, Republic of Korea; 7 Department of Chemical and Biomolecular Engineering, Yonsei University, Seoul, 120-749, Republic of Korea

## Abstract

We report that a single crystal Ni_2_Si nanowire (NW) of intermetallic compound can be reliably designed using simple three-step processes: casting a ternary Cu-Ni-Si alloy, nucleate and growth of Ni_2_Si NWs as embedded in the alloy matrix via designing discontinuous precipitation (DP) of Ni_2_Si nanoparticles and thermal aging, and finally chemical etching to decouple the Ni_2_Si NWs from the alloy matrix. By direct application of uniaxial tensile tests to the Ni_2_Si NW we characterize its mechanical properties, which were rarely reported in previous literatures. Using integrated studies of first principles density functional theory (DFT) calculations, high-resolution transmission electron microscopy (HRTEM), and energy-dispersive X-ray spectroscopy (EDX) we accurately validate the experimental measurements. Our results indicate that our simple three-step method enables to design brittle Ni_2_Si NW with high tensile strength of 3.0 GPa and elastic modulus of 60.6 GPa. We propose that the systematic methodology pursued in this paper significantly contributes to opening innovative processes to design various kinds of low dimensional nanomaterials leading to advancement of frontiers in nanotechnology and related industry sectors.

Nanotechnology plays a key role in advancing the frontiers of sciences and industries through innovative breakthroughs to conventional limitations defined by theories and rules for macroscopic counterparts. Nanomaterials for bio-mimetic application, high-performance semiconductors and nanoelectromechanical systems (NEMS) are the archetype examples. Many of the functionality are unique for nano-scale materials and seldom available with bulk counterparts. In general, materials properties of nanoscale regime are substantially dependent on the control level of processing nanomaterials, which is typically by far more complicate than bulk. This situation becomes even more challenging as material dimension reduces to one (quantum dot) or two (nanowire)^1^,^2^.

Nanowires (NWs) have been widely employed as key components for electric circuits^3^, optical nanodevices^4^ and so on. Especially, Si-based NWs are indispensable for various kinds of devices exposed to high mechanical loadings and electric or chemical perturbations^5^. In such circumstances, the NWs should possess (electro-) chemical stability and mechanical integrity over long-term operation^6^,^7^,^8^,^9^. Intermetallic compounds of silicide Ni_2_Si NWs occupies unique position in such a case due to its favorable properties, and has a wide range of applications as interconnectors of semiconductor devices^10^, ohmic contacts^11^, and gate materials of integrated circuit (IC) chips^12^.

While electric properties of the Ni_2_Si NWs have been extensively studies by theories and experiments^13^,^14^,^15^, mechanical behaviors were still inferred from indirect measurements on NW samples or extrapolated from bulk materials, largely due to difficulties in reliable design and experimental test of NW specimen. Both of the electric and mechanical properties of a Si-based NW have to be well characterized to further spread out its application and commercialization areas. In this study, we systematically developed innovative design method to simply but reliably design Ni_2_Si NWs of micrometer lengths. To accurately measure mechanical properties we directly applied tensile tests on the Ni_2_Si NWs and pursued mechanistic understanding by extensive utilization of DFT calculations. Structure-mechanical properties relationship was further validated with HRTEM^16^,^17^,^18^, EDX observations.

Conventionally, NWs have been designed with so-called bottom-up approach^6^,^13^,^14^,^15^, which scales up individual atoms or clusters into nanomaterials with the chemical vapor deposition (CVD) process followed by crystal growth in liquid or gas phase. Then, final form of NWs in such process is completed in a template. Differently from the traditional method, it is noteworthy that Bei *et al*.^7^,^8^,^9^ obtained single crystal Mo NWs by chemically extracting micrometer pillars. The key idea in the report was to utilize phase transformation caused by eutectic reaction in the alloy matrix. This study, indeed, opened new doors for designing NWs based on phase separating solid-state reactions such as eutectoid and precipitation^19^. Eutectoid reaction transform one solid into two solid phases (α → β + γ), while in the discontinuous precipitation secondary phases are generated or grown from matrix material α (α → α + γ or α → α’ + γ). Therefore, the structure of the matrix would be maintained after the discontinuous precipitation but the compositions of elements in the precipitates decrease in the solid matrix. Consequently, the difference of the two mechanisms is whether the initial phases can be preserved or not via the phase transformation. The driving force for the phase transformation of the discontinuous precipitation is much lower than that of the eutectic reaction. Thus, the driving force of the discontinuous precipitation is more sensitive to the interfacial energies between the secondary phases and matrix, dominantly growing facets of low interfacial energies at the expense of high index planes. The facets of the precipitates and matrix at the interfaces are thermodynamically stable and coherent, which is useful for controlling thicknesses (or diameters) of precipitates. Consequently, we could make nanopillars as thin as one tenth of pillars obtained by typical eutectic reactions^20^.

Unlike conventional methodology, we propose that discontinuous precipitation (DP) process can simplify complicate design steps for designing NWs, especially for intermetallic compounds. The DP has been widely applied to make precipitates in alloy metal and generally neglected for the purposes of enhancing mechanical properties since the size and aspect ratio of precipitated particles are rather too large. Anisotropic nature of interfacial energy and strong strain field between the precipitates and matrix were known as the origin^21^,^22^.

In this paper, however, we discovered that the DP method could be useful for simple design of intermetallic silicide NWs with well-controlled mechanical properties as desired.

## Results and Discussion

**Figure 1:**
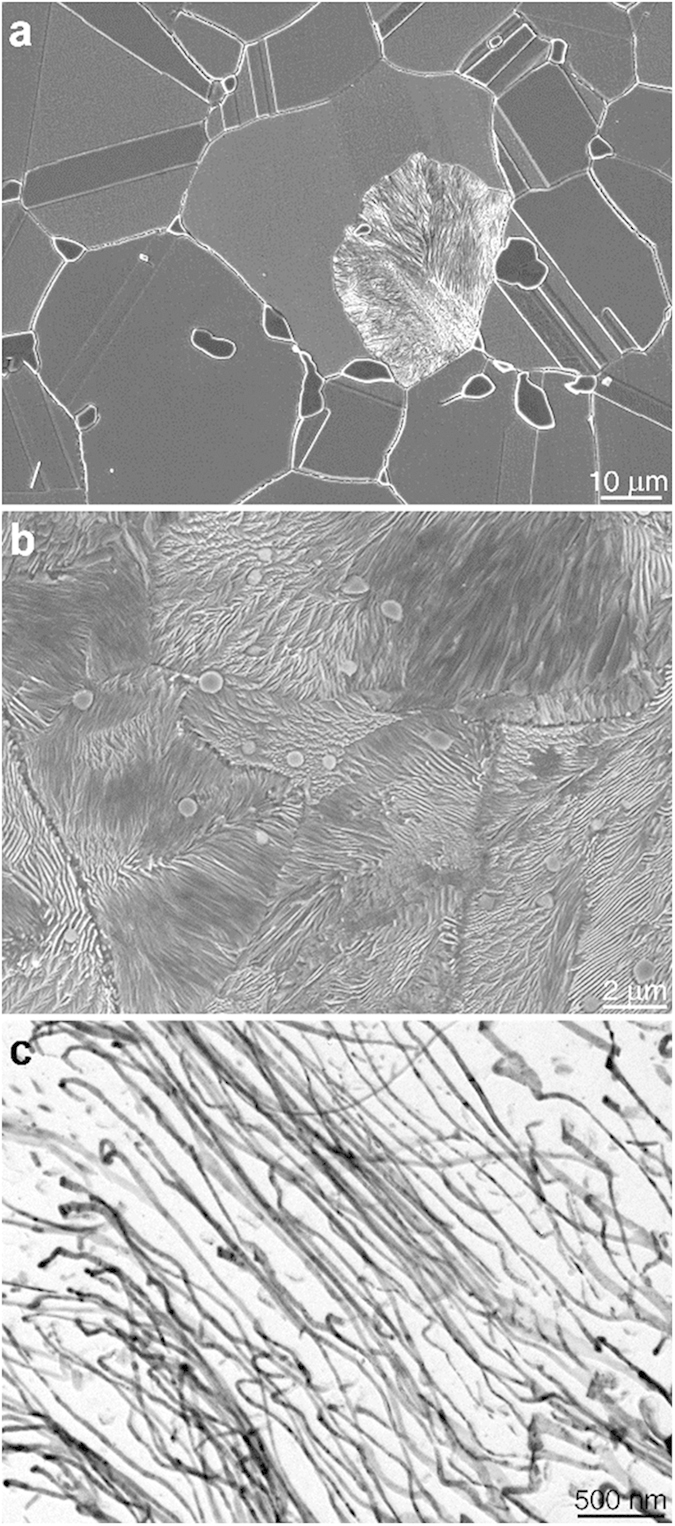
SEM images of a Cu-6Ni-1.5Si alloy matrix and Ni_2_Si nanowire. Discontinuous precipitations of Ni_2_Si nanoparticles in the grains of Cu alloy matrix (the grey-colored area) in (**a**) was appeared after 1 hour of heat treatment at 980 °C, followed by half an hour of thermal aging at 500 °C. The DP was fully developed after 7 hours of thermal aging as shown at (**b**). Image (**c**) depicts Ni_2_Si nanowires decoupled from the Cu-6Ni-1.5Si matrix using chemical etching process with conventional acids of NHO_3_ and C_2_H_5_OH with each 50 ml.

Our model system was Cu-6Ni-1.5Si alloy and applied three major steps to obtain complete Ni_2_Si NWs. To facilitate formation DPs of Ni_2_Si nanoparticles we added 0.1 wt.% of Ti to the model alloy system and carried out heat treatments^21^ on the solid solution at 980 °C. The grey area in Fig. 1a shows a SEM image of the grains of Cu-6Ni-1.5Si alloy, where the initial DPs appeared after thermal aging at 500 °C for a half an hour. After seven hours of thermal aging DPs were fully developed over the entire alloy as shown at Fig. 1b. To scale up the nanoparticle DPs up to micrometer length Ni_2_Si NWs, we thermally elongated the DP shown at Fig. 1b. And then, we chemically etched the Cu-6Ni-1.5Si alloy in conventional acidic solution composed of NHO_3_ and C_2_H_5_OH with each 50 ml to decouple Ni_2_Si NWs as deposits in the solution. Figure 1c represents the SEM image of the Ni_2_Si NWs.

**Figure 2:**
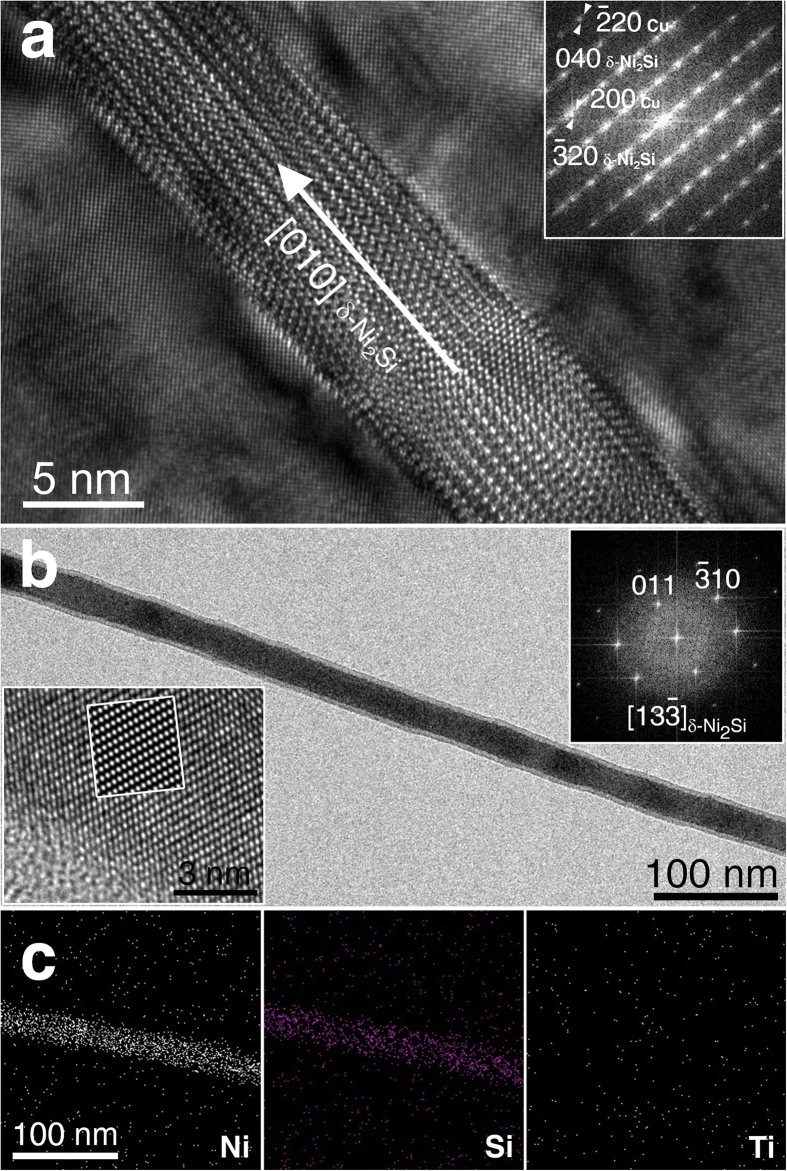
High resolution TEM images of DP region in the grain of Cu-6Ni-1.5Si matrix in (a) and in (b) a monolithic single crystal of Ni_2_Si nanowire. Image (c) shows EDX analysis confirming compositions of Ni_2_Si nanowire of 66.6 at% Ni, 33 at% Si and no Ti.

**Figure 3:**
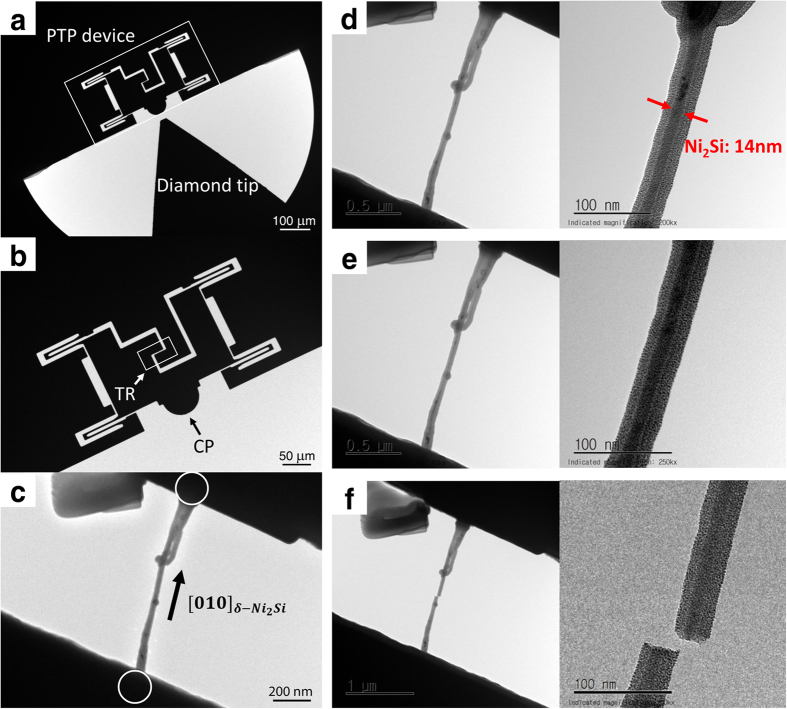
Schematic pictures of the tensile tests on a Ni_2_Si nanowire with a push-to-pull (PTP) device. The PTP device and a diamond tip were attached to the force transducer as shown in (**a**) and in (**b**) the enlarged image of the PTP device and a tip contact point (CP) and a tensile region (TR, marked by a rectangle) were represented. A Ni_2_Si nanowire sample mounted to the ends of the TR by Pt deposits (marked by circles) was shown in (**c**). The captured TEM photographs of the Ni_2_Si nanowire were provided for three major moments of the tensile test: before in (**d**) during in (**e**), and after mechanical fracture at (**f**).

Our HRTEM observed that the Ni_2_Si NWs are monolithic structures with average diameter of 13.7 nm and 10 μm in length. Considering that theoretical weight (0.075) and volume (0.085) fractions it implies that we enabled to design approximately 750 g of Ni_2_Si NWs from 10 kg of Cu-6Ni-1.5Si alloy using our process. Surprisingly, both HRTEM and EDX (Fig. 2) clearly showed that the Ni_2_Si NWs are δ-phase structures of a single crystal uniformly well extended in only [010] direction, in spite of the brittle nature as an intermetallic compound. Figure 2b shows bright field of TEM and HRTEM images of the Ni_2_Si NWs. The monolithic structure drawn from the Ni_2_Si NWs along the [13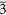] zone axis was shown at the inset in Fig. 2b (at upper right part). The simulated images of TEM for the Ni_2_Si along the [13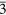] zone axis were also added at left part of the HRTEM image in Fig. 3b, which is consistent with the experimental observation on 6.0 nm thick specimen by a defocus value of Δf = −29.0 nm. Images obtained from the computer simulations with thicknesses ranging from 5.0 to 20.0 nm by defocus values between −20.0 and −40.0 nm. Most interestingly, the HRTEM analysis revealed that the interfaces between the alloy matrix and the embedded Ni_2_Si NW were almost in full coherency^23^,^24^ with only slight lattice misfits (Δ). For instance, 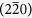
_matrix_//(040)_Ni2Si NW_ (Δ ≈ 0.0245, 0°), (020)_matrix_//(320)_Ni2Si NW_ (Δ ≈ 0.0554, 1.67°) and (001)_matrix_//(001)_Ni2Si NW_. Such tightly coherent interfaces would force DPs of Ni_2_Si nanoparticles to grow into anisotropic morphology (i.e., discontinuous cellular shape) during thermal aging, which was not the case of our sample.

As far as we know, direct tensile tests on intermetallic NW compounds were rarely reported previously due to a tremendous difficulty in making and testing a NW sample. Thus, mechanical properties of intermetallic NWs were rather extrapolated from bulk counterpart properties measured by compressive loadings.

Figure 3 illustrates the PI95 stage for the direct tensile on the Ni_2_Si NW sample processed by our three-step process. The PI95 stage was equipped with a force transducer to control the position of mounted diamond tip and to measure applied force at the tip. When the tip contact point (CP) of the push-to-pull (PTP) device is moved by the position controlled diamond tip, the distance between the tensile regions (TR) of the device increases (marked by a rectangle in Fig. 3b). Figure 3c shows a Ni_2_Si NW sample for the tensile test with Pt at both ends of the tensile region (TR) in PTP device (marked by circles). We deposited Pt using an electron beam induced gas deposition technique^25^ to firmly mount the NW specimen. The gas was supplied with a gas injection system (GIS) in the FIB. The yield strength of pure Pt (about 200 ~ 300 MPa) is far below typical values of intermetallic compounds. Pt also has much higher ductile than intermetallic Ni_2_Si. Consequently, it is reasonable to assume that experimentally measured stress and strain behaviors of the Pt-deposited Ni_2_Si nanowire well characterize the intrinsic Ni_2_Si nanowire.

**Figure 4:**
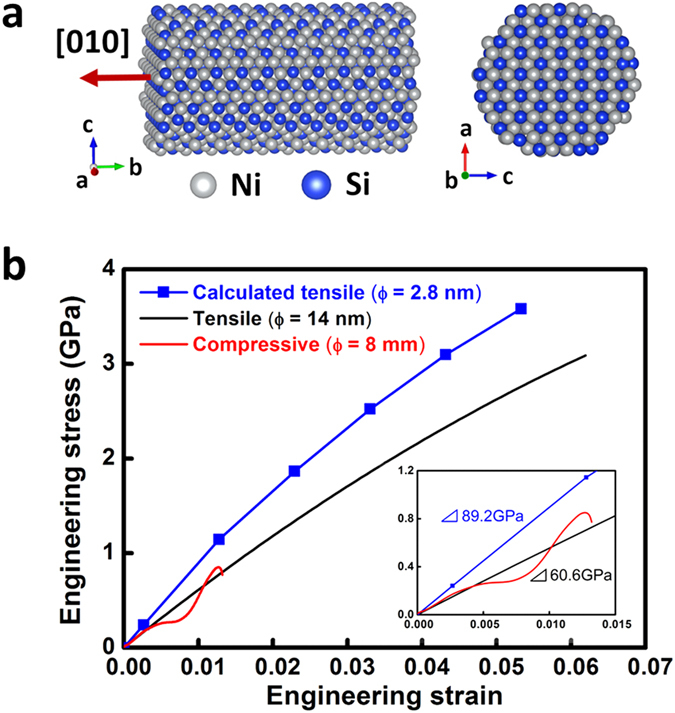
DFT model system simulating Ni_2_Si nanowire in (a) and in (b) stress versus strain behaviors measured by tensile tests (the black line) and calculated by first principles DFT computings (the blue line) in the [010] direction of the Ni_2_Si nanowire. The red line in (**b**) shows experimental results for a bulk Ni_2_Si intermetallic compound (8 mm in diameter and 10 mm in length) tested by loading compressive stress. The inset depicts the stress response at engineering strains less than 1.5 % before significant fluctuations appeared due to the formation and growth of cracks in the bulk Ni_2_Si sample.

We also effectively removed the influence of Pt on mechanical properties of the Ni_2_Si NW by following two rational procedures. First, we measured yield strength of a *bulk* Ni_2_Si intermetallic compound by imposing compressive strains noticed as the reddish curve in Fig. 4 (details are described at next paragraph). The measured value of 0.9 GPa was considerably higher than the yield stress of bulk Pt (30 MPa). Secondly, we noticed that the measured elastic moduli measured by a tensile test on Ni_2_Si NW with Pt deposits and by compressive test on pure bulk Ni_2_Si are fairly similar as shown at Fig. 4. These facts provide reasonable ground for the assumption that Pt deposits are not critical in measuring mechanical properties of Ni_2_Si NW sample only.

Tensile stress was loaded to the [010] direction of a Ni_2_Si NW sample with a strain rate of 0.001 s^−1^. Using the known spring constant of PTP device we converted the measured load vs. strain relationship into stress vs. engineering strain curve as shown at Fig. 4. We neglected the strain regions of less than 1.7 % since Ni_2_Si NW was not yet completely aligned in the [010] tensile direction as shown at Fig. S1. Our tests showed that tensile strength and elastic modulus of a Ni_2_Si NW are approximately 3.0 and 60.6 GPa, respectively.

We performed compressive stress-strain tests for in-house made a bulk polycrystalline Ni_2_Si intermetallic compound made by a vacuum arc melting method. Measured yield strength and strain on the bulk Ni_2_Si polycrystal were 0.9 GPa and 1.25 %, respectively, which is within values of typical brittle materials. Mechanical hardness was measured as 620 Hv by a Vickers hardness tester with 20 g loading and 20 seconds of dwelling time, and it can be roughly converted to tensile strength of 2.07 GPa. Thus, the tensile strain (6 %) of the single crystalline Ni_2_Si NW shown at Fig. 4 is much higher than that of polycrystalline bulk counterpart, even though both fracture by as typical behaviors of brittle materials (i.e., no noticeable necking until the strain reaches the mechanical fracture level). The bulk modulus of the polycrystalline Ni_2_Si measured at early stage of the strains before microcracks initiate or propagate (the inset at Fig. 4) is similar to that of a single crystalline Ni_2_Si NW. Consequently, it implies that the bulk modulus of the single crystalline Ni_2_Si NW in this experiment is reliable since it is essentially related to atomic bond strength of a material. Moreover, it also confirms that the Pt layer (amorphous structure) negligibly influences on the mechanical properties of the Ni_2_Si NW.

In summary, we quantitatively characterized the mechanical behaviors of δ-phase Ni_2_Si NWs through the direct uniaxial tensile tests, which has been rarely reported in previous literatures.

Using first principles DFT calculations we calculated the stress vs. strain behaviors of Ni_2_Si NW. Figure 4a illustrates the computational model system simulating Ni_2_Si NW, which are composed of 180, 90 of Ni and Si atoms, respectively. The sizes of Ni_2_Si NW model are 2.80, 0.49 nm in diameter (the *a*-axis of the model system) and length along the longitudinal direction ([010], the *b*-axis), respectively. We imposed a tensile strain with a rate of 1 % into the *b*-axis direction at each step. Elastic modulus, *E*, of the Ni_2_Si NW was calculated by


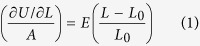


where *U*, *L* (*L*_*0*_ = 4.927 Å), *A* indicate that total energy calculated by DFT computing, the (initial) length, and cross-sectional area of the Ni_2_Si NW model system, respectively. Since the surface layer of our NW model is atomically rough we estimated the *A* from the volume (*V*) by


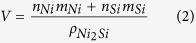


Here *n*, *m*, and *ρ* indicate the number of atoms, atomic mass and density, respectively. Based on experimental literatures^26^,^27^. We used 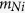 = 58.342 gmol^−1^, 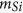 = 28.085 gmol^−1^ and 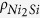 = 7.2 gcm^−3^, and identified *V* = 3005.003 Å^3^ and thus, *A* at each tensile strain was easily calculated by *V*/*L*. We plot the calculated stress vs. strain behaviors of the Ni_2_Si NW at Fig. 4b together with the experimental measurements. Even though the detailed morphology and size of the model system are not completely the same as the experimental NW sample the overall behaviors are fairly in good agreements. DFT calculations seem to slightly overestimate the experimental results. It may be ascribed to atomic defects in the experimental Ni_2_Si NW sample, while the DFT model system is a perfect crystal. It is reasonable that surface or internal defects should decrease mechanical strength of a NW since they could work for active sites of nucleating or aiding growth of microcracks^28^. The influence of the defects would be conspicuous as tensile strain increases, as well represented by the larger deviations between DFT calculations from experimental measurements at higher strain regions shown at Fig. 4b.

## Conclusions

In conclusion, we demonstrated that a single crystalline Ni_2_Si NW of an intermetallic compound could be easily designed by a three-step method: the casting alloy with proper compositions to create DP nanoparticles in the grains of the alloy matrix, scaling up the nanoparticle DPs into nanowire with micrometer lengths using thermal elongation, and lastly the chemical etching of the whole system to decouple NWs from the matrix. This approach enabled us to design Ni_2_Si NWs with uniform morphology and composition over several μm in lengths. Direct uniaxial tensile tests on the NW integrated with HRTEM and EDX, and first principles DFT calculations provided consistent structure-mechanical property relationship on the Ni_2_Si NW. It was clearly provided that the tensile strain of a Ni_2_Si NW is much higher than bulky counterpart in spite both showed the fracture natures of brittle materials. Our methodology will contribute to paving new ways to easy manufacture and design of brittle NW materials with large aspect ratio, potentially leading to opening the application of nanotechnology wider industrial sectors.

## Methods

### Design of Materials

Pure copper and silicon of 99.99 % purity, nickel of 99.9 % purity and titanium of 99.8 % purity were used for design of the Ni_2_Si NWs. The weight fraction of each alloy element was 6.2 %, 1.34 Si, and 0.11 for Ni, Si, and Ti, respectively with a Cu as the base material. Cu-Ni-Si-Ti alloy ingot of 20 mm thickness was designed by vacuum induction melting, and rolled at 980 °C until the thickness wad reduced to 6 mm. To eliminate the thermo-mechanical history of the specimen the hot-rolled plates subsequently passed a heat treatment at 980 °C for 1 hours, followed by thermal aging for 7 hours at the same temperature. The aged alloy was, then, dipped into acidic solution composed of NHO_3_ and C_2_H_5_OH with each 50 ml to decouple Ni_2_Si NW from the Cu alloy matrix. The wires were cleaned by ultrasonic treatment in pure ethanol for 10 minute.

### TEM experimental method

Microstructural characterization of Ni_2_Si NWs was performed by 200 kV field-emission TEM using a Jeol JEM-2100F equipped a scanning TEM (STEM) with EDS. To characterize mechanical properties of Ni_2_Si NWs we directly imposed tensile strains with a low rate along its longitudinal direction at ambient temperature until it fractures. Whole of the procedure was recorded by Orius SC200D CCD camera (Gatan) and Virtual Dub software with a frame rate of 1 fps (frame per second). Tensile tests were conducted in a JEOL 2100 LaB_6_ TEM using a Hysitron’s Picoindentor specimen stage (PI95) with a MEMS-based push-to-pull (PTP) device as shown at Fig. 3.

### Computational details

All DFT calculations were carried out using the Vienna Ab-initio Simulation Package (VASP)^29^ with the Projector Augmented Wave (PAW)^30^ pseudo-potentials. We used the Perdew-Burke-Ernzerhof (PBE)^31^ exchange-correlation functional. The cutoff energy of the plane wave basis was 300 eV. We integrated the Brillouin zone with a gamma point scheme of 1 × 3 × 1 *k*-points. The δ-Ni_2_Si NW was modeled by cylindrically shaped structure with a 2.8 nm diameter. Supercells included Ni_2_Si nanowire composed of 90, 180 of Si and Ni atoms, respectively, truncated with (010) facet through the b-axis illustrated in Fig. 4a. To preclude any interaction of Ni_2_Si with its images we inserted 1.5 nm of vacuum space in the transverse direction of the nanowire.

## Additional Information

**How to cite this article**: Han, S. Z. *et al*. Reliable and cost effective design of intermetallic Ni_2_Si nanowires and direct characterization of its mechanical properties. *Sci. Rep*. **5**, 15050; doi: 10.1038/srep15050 (2015).

## Supplementary Material

Supplementary Information
