# Supplementary material for: Reliable and cost effective design of intermetallic Ni2Si nanowires and direct characterization of its mechanical properties
Source: Sci Rep. 2015 Oct 12;5:15050. doi: 10.1038/srep15050 (PMC4601013; doi:10.1038/srep15050)
Supplement: Supplementary Information [file srep15050-s1.pdf]

**Supplementary Information for:**

# Reliable and cost effective design of intermetallic $Ni_2Si$ nanowires and direct characterization of its mechanical properties

*Seung Zeon Han[1], Joonhee Kang[2], Sung-Dae Kim[1], Si-Young Choi[1], HyungGiun Kim[3], Jehyun Lee[4], Kwangho Kim[5], Sung Hwan Lim[6]*, and Byungchan Han[7]**

[1]Structural Materials Division, Korea Institute of Materials Science, Changwon 642-831, Republic of Korea

[2]Department of Energy Systems Engineering, DGIST, Daegu, 711-873, Republic of Korea

[3]Gangwon Regional Division, Korea Institute of Industrial Technology, Gangneung 210-340, Republic of Korea

[4]Department of Materials Science and Engineering, Changwon National University, Changwon 641-773, Republic of Korea

[5]School of Materials Science and Engineering, Pusan National University, Busan, 609-735, Republic of Korea

[6]Department of Advanced Materials Science and Engineering, Kangwon National University, Chuncheon 200-701, Republic of Korea

[7]Department of Chemical and Biomolecular Engineering, Yonsei University, Seoul, 120-749, Republic of Korea

*Corresponding authors. E-mail: shlim@kangwon.ac.kr, bchan@yonsei.ac.kr

**In-situ tensile test of $Ni_2Si$ intermetallic compound wire**

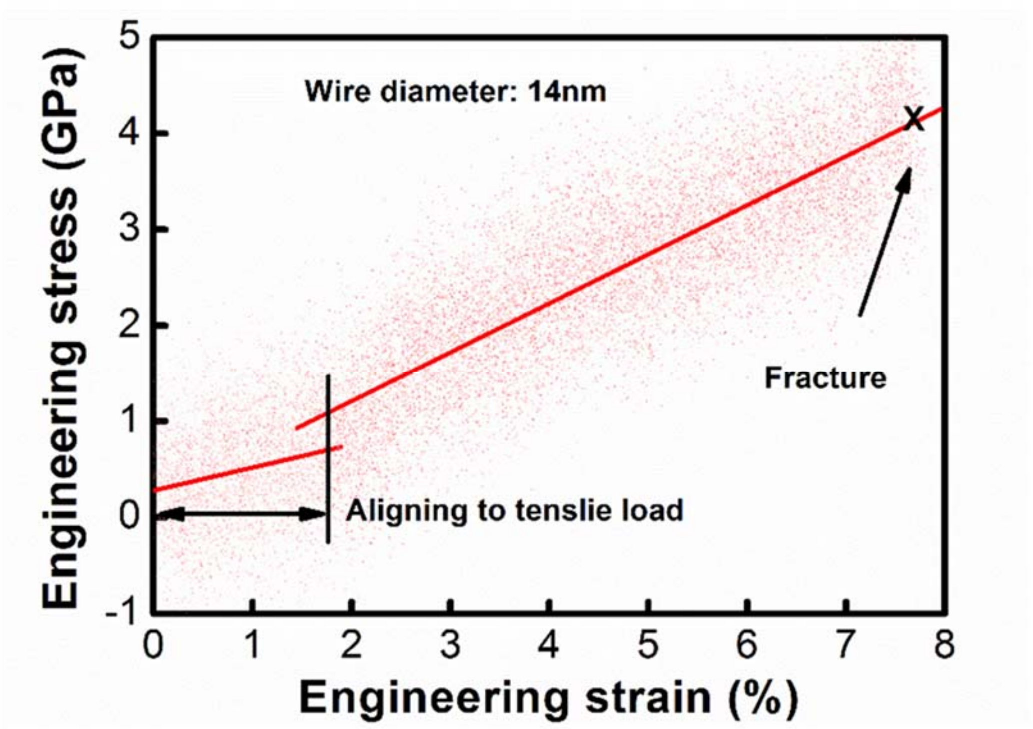


**Figure S1.** Stress vs. strain relationship less than 1.7 % of the engineering strain was not considered to obtain the mechanical behaviors since in this region the $Ni_2Si$ nanowire was not yet fully aligned in the [010] tensile direction.
